# Erdheim–Chester Disease of the Breast: First Review and First Case of Isolated Severe Gynecomastia

**DOI:** 10.3390/diagnostics13071239

**Published:** 2023-03-25

**Authors:** Francesco Ruben Giardino, Roberto Cuomo, Mirco Pozzi, Gianluca Marcaccini, Stefano Bacchini, Mohamed Marzouk El Araby, Luca Grimaldi, Giuseppe Nisi

**Affiliations:** Surgery and Neuroscience—Plastic Surgery Unit, Department of Medicine, University of Siena, Policlinico Santa Maria “Le Scotte”, 53100 Siena, Italy

**Keywords:** Erdheim–Chester disease, gynecomastia, histiocytosis x, mastectomy, breast disease

## Abstract

(1) Introduction: Erdheim–Chester disease (ECD) is a life-threatening condition and often a diagnostic challenge. It has recently been classified as a hematopoietic tumour, and the cases of ECD reported in the literature has dramatically increased during the last 15 years. (2) Methods: We describe the case of a 57-year-old male patient with severe gynecomastia, with a detailed description of his diagnostic *iter* and consequent surgical operation. We provide the first systematic review of the literature of breast involvement in ECD, following PRISMA guidelines, including 13 studies and 16 patients. (3) Results: Our report resulted to be the first case of gynecomastia as a single clinical and imaging feature of ECD described in English literature. A total of 81.3% of patients included were female. Among them, 76.9% had unilateral and nodular presentation, while male patients presented bilateral heterogeneous breast enlargement. Globally, 87.5% expressed breast alterations as their first manifestations of ECD. Only 50% presented skeletal involvement. (4) Conclusion: The reported case represents a unique addition to the literature. We found two different patterns in ECD-related breast involvement between male and female patients, an unusual M/F ratio, and a lower rate of bone involvement. Breast involvement is frequently the first clinical feature; therefore, breast caregivers should be aware of this dangerous and most likely underestimated condition.

## 1. Introduction

The Erdheim–Chester disease (ECD) is a dangerous and rare type of histiocytosis, firstly described by William Chester and Jakob Erdheim in 1930 as a lipoid granulomatosis, while the first reported case on PubMed is dated 1978 [1]. Since then, less than 1000 cases have been reported in the global literature, with a wide spectrum of symptoms related only to its multi-organ involvement (e.g., papilledema, hypogonadism, hypopituitarism, Horner syndrome, testicular pain, ataxia, scalp nodules, renal artery stenosis).

Its pathogenesis is currently still poorly understood. Finding BRAF v600e mutation in more than 50% of ECD patients [2] has led to the formulation of a clonal neoplastic origin for this disease, and the clinical efficacy of Vemurafenib (V600E-mutated BRAF protein inhibitor) in patients who harbour this mutation seems to confirm this theory [3,4].

On the other hand, the evidence of a dominant Th1 immune response (due to low serum IL-4 levels, and high IFN-alpha, IL-7, IL-12 levels) and the therapeutic efficacy of alpha-interferon, anakinra [5,6], infliximab [7], and steroids [8,9] seem to be consistent with a dysregulated inflammatory pattern.

While the pathogenesis is unclear, it is widely accepted that the diagnosis is based on the detection of migration and infiltration of lipid laden CD68+/Cd1a(-) histiocytes, which may occur in different tissues [10], resulting in fibrosis and even deep modifications of the target organ’s anatomy, with a variable grade of function impairment.

Different affected areas may express different signs and symptoms, and this may result in several patterns of clinical presentation.

Skeleton involvement is the most common type (PET/CT are commonly performed in patients suspected for ECD) as it appears in 96% of patients [11], but many other organs can also be involved, such as: the central nervous system [12,13], cardiovascular system [14,15], lungs [16], kidneys [17], adrenal glands (and a great variety of endocrine manifestations as well) [18], skin [19], gastrointestinal tract [20,21,22], skeletal muscle [23,24,25,26], and thyroid [27].

Hemophagocytosis has also been reported [28]. Before the introduction of IFN-alpha in the treatment of ECD, the mean survival after diagnosis was calculated to be 19.2 months. Today, the mortality rate is 26%, and a five-year survival rate is 68%.

Breast involvement is currently considered rare, with no percentages available in the current literature, and with barely two other cases reported in men [29,30].

ECD is currently included in the 2016 World Health Organisation (WHO) classification of hematopoietic tumours [31], and the diagnosis has been confirmed through the discovery of CD68+, CD163+, FXIIIa+, and CD1a- cells corresponding to “non-LCH” histiocytes, which has been grouped among the “L” (Langerhans) group of the 2016 revised histiocytosis classification of the Histiocyte Society [32].

## 2. Materials and Methods

### 2.1. Case Study

**Figure 1:**
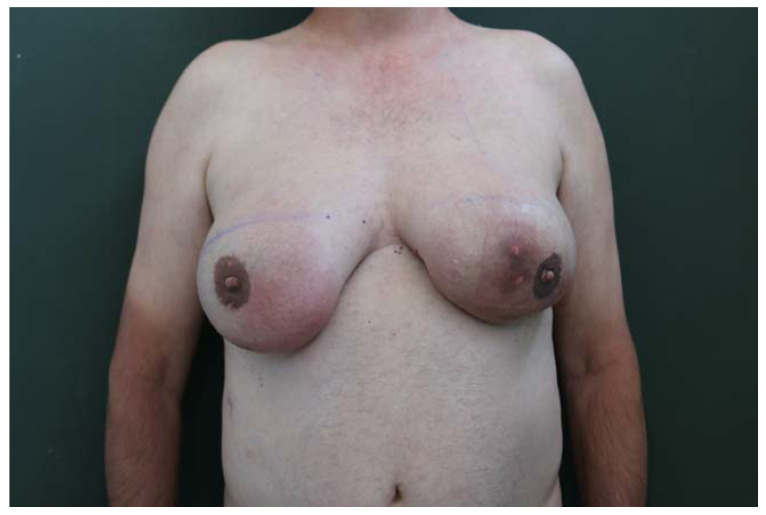
Grade 4 bilateral gynecomastia (according to Rohrich classification) with an asymmetric development of the glands.

A.G., male and 57 years old, came to our unit of plastic and reconstructive surgery presenting a severe bilateral gynaecomastia (Figure 1).

From the age of 50, he had noticed an increase in the volume of his breasts, describing the rate of growth as being faster during the first two years, and slower, but progressive, in the remaining five years. 

**Figure 2:**
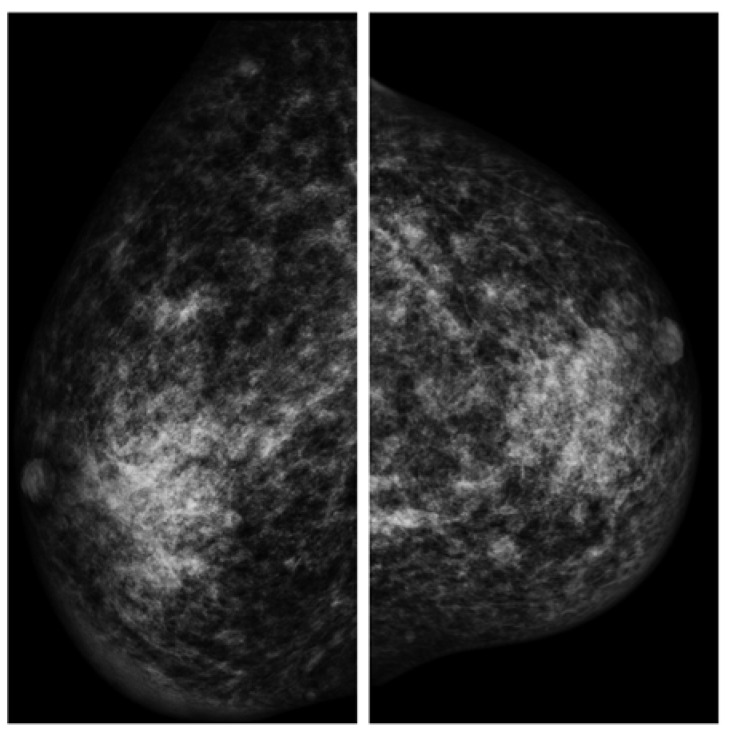
Mammography findings (left and right breast).

The patient underwent mammography, mammary ultrasound, agobiopsy (TRU-CUT), and serum exams in order to examine his clinical condition from 2015 to 2016 (Figure 2).

The malignant nature of the tissue was excluded, since no suspicious calcifications came to evidence in the mammary glands, but the presence of hemosiderinic pigment was confirmed by agobiopsy. No hormonal abnormalities were detected (the levels of prolactine, beta-HCG, and estradiol were within the expected range in three different exams).

Several mammary simple cysts, with diameters ranging between 5 and 30 mm, suggestive of fibroadenomas, and a dense fibroglandular breast parenchyma were reported by the radiologist, who described these radiological findings as uneven in a “normal” gynecomastia.

In 2017, the patient was committed to our unit for this unusual gynecomastia, and during the pre-operative imaging exams of the chest and a further bioptical pulmonary exam, a squamous cell carcinoma on the right upper lobe of the lung was incidentally discovered (G2ypT1bN1).

Four cycles of chemotherapy with gemcitabine, vinorelbine, and cisplatin-based neoadjuvant chemotherapy were set up and the progression of gynecomastia stopped during therapy. At this point, a paraneoplastic nature of the gynecomastia seemed to have been the most probable diagnosis to endocrinologists, thoracic surgeons, and the general practitioner, due to a single increase in prolactin levels (25.6 pg/mL; normal range: 2–13 pg/mL) found in 2017 during pre-operative serum exams, and due to the concomitant lung cancer. Nevertheless, the real nature of the condition was still unclear. A lobectomy of the right upper lobe was performed in November 2017, followed by adjuvant chemotherapy, which led the patient to heal from cancer. Finally, the patient returned to our care for the treatment of a paraneoplastic gynecomastia. 

Upon clinical examination, the man’s breasts presented a grade 4 bilateral gynecomastia (according to Rohrich et al.) [33] with an asymmetric development of the glands (Figure 1) and hyperpigmentation of the involved skin areas. Jugulum–NAC distance was measured (17 cm on the right breast; 17.5 cm on the left breast). A high circumareolar skin tension was detectable, in particular on the left breast, where a cutaneous ulceration was evident. On breast palpation, a hard consistency of the glands and a parenchymal nodule in the external upper quadrants (about 2 cm) were detectable. 

Finally, neither mastodynia, nor inducible or spontaneous galactorrhoea was evident. 

The patient expressed a strong psychological discomfort with regard to his severe chest feminisation, along with a high-grade of social and sexual impairment.

We scheduled a subcutaneous mastectomy with a free NAC graft.

**Figure 3:**
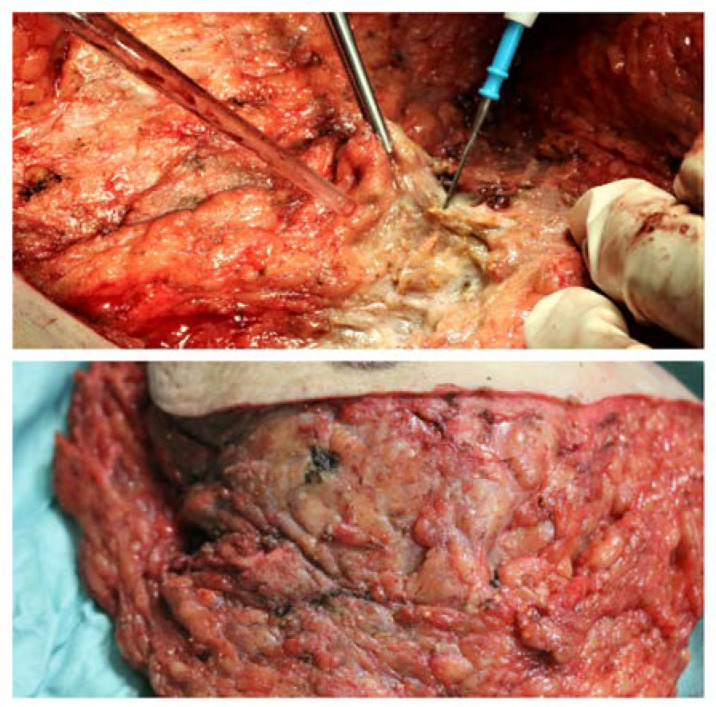
Intraoperative findings: (**Above**) The mass was deeply attached to the pectoralis major muscle fascia, and so it was difficult to identify a clean cleavage plan. An atypical appearance of mammary glands, partially indistinguishable from fat tissue, resulting in a single tough, dense mass with several small (<1 cm) orange calcified concretions, and fat necrosis areas. (**Below**) An atypical appearance of mammary glands, partially indistinguishable from fat tissue, resulting in a single dense mass with small orange calcified concretions, and fat necrosis areas as well.

An atypical macroscopic appearance of the mammary glands, partially indistinguishable from fat tissue, and resulting in a tough, dense mass with several small (<1 cm), orange calcified concretions, together with areas of fat necrosis, were found intraoperatively. The mass resulted closely attached to *pectoralis major* muscle fascia and to the skin and subcutaneous tissue, and this made a clean surgical dissection very hard to perform (Figure 3).

**Figure 4:**
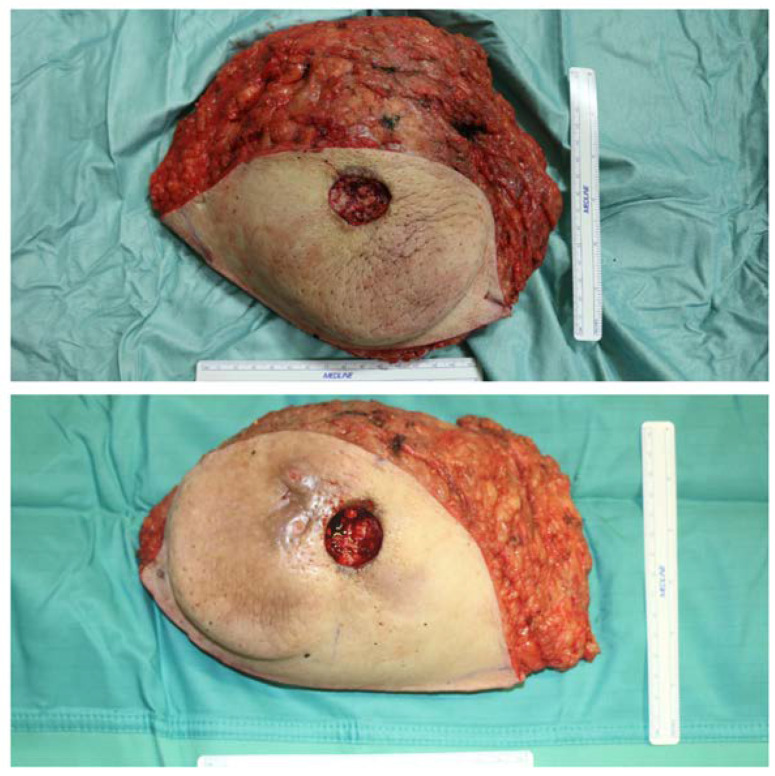
Appearance of excised breasts; size compared to a 15 cm measuring stick.

Skin excess and both mammary masses were removed en bloc, approximately 1480 g from the left side and 1240 g from the right side (Figure 4) and, based on the intraoperative and previous histological findings of inflammatory reactions in 2016, the tissues were sent for further histological and immunohistochemical analyses.

Two Redon suction drains were placed and the patient was kept under observation for two days, renewing the compressive dressings once a day.

The patient was then dismissed and evaluated after a week, and is nowadays in follow-up (last postoperative control was performed 12 months after the operation).

Given the high probability of bone involvement in the syndrome, we considered it necessary to investigate the skeletal system through a positron emission tomography, which is able to determine the spreading and the activity of the disease by conducting a whole body assessment of all lesions in a single session. In addition, it is superior to other imaging modalities [34]. In particular, technetium DPD is specific in the detection of bone anomalies.

Total body CT scans performed for lung cancer staging were re-evaluated by the radiologists in order to detect other abnormalities related to the ECD once it was confirmed.

The typical molecular pattern of this particular type of histiocytosis (BRAF v600e) was also investigated.

Based on the peculiar clinical features of our patient, we performed a comprehensive systematic review, including studies that reported cases of Erdheim–Chester disease with breast involvement.

### 2.2. Systematic Review

#### 2.2.1. Study Selection 

Following the preferred reporting items for systematic reviews and meta-analyses guidelines [35], the review included reports of ECD involving the breast, in both male and female patients. Studies were included if they met the following criteria: manuscripts were case reports or series, or described a case report in the text; cases were histologically diagnosed with ECD with breast involvement (regardless of the sex), and studies were written and published in English. Studies were excluded if they were literature reviews, or at least without a description of a case report. 

#### 2.2.2. Variables of Interest

Data from the studies selected included: author, age, sex, location, breast features, breast involvement as the first sign of ECD, long bones involvement, relevant clinical history/features.

#### 2.2.3. Data Sources and Search Strategy

An extensive review was conducted on 1 October 2022, for all articles including Erdheim–Chester disease with breast involvement, and the research was performed in 2 different databases: PubMed and Medline. The keywords for the research strategy were “Erdheim–Chester” AND (“mammary glands” OR “mammary”) OR “Erdheim–Chester” AND (“breast” OR “breasts”) OR Erdheim–Chester AND (“gynecomastia” OR “gynaecomastia”). The cases were selected by 2 independent reviewers, in a 2-step inclusion process: step 1: review of title and abstract (or summary) and step 2: a full-text review.

## 3. Results

The patient returned for an early postoperative check one week later, showing good clinical conditions. Signs of ischemic suffering of both NACs were evident, while the inframammary wound was in a satisfactory state after an appropriate wound-care and management of NAC’s skin necrosis [36,37,38,39,40].

**Figure 5:**
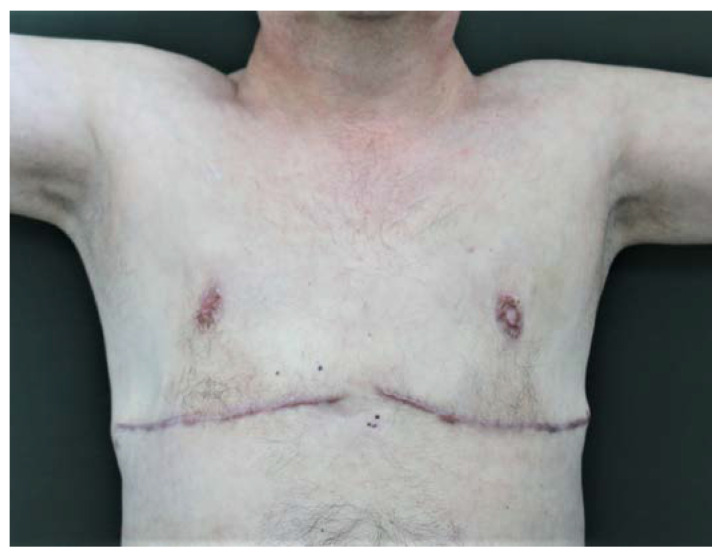
The sixth-month postoperative clinical follow-up.

The patient is still in follow-up after our surgery, has no further alterations to his clinical setup, and presents the cicatricial remnants of a bilateral nipple necrosis, which, however, did not affect the evident improvement of the quality of his life (Figure 5). Total body CT scans were negative for radiological signs such as hairy kidneys, coated aorta, pericardial fibrosis, and interstitial lung involvement.

The positron emission tomography/CT with technetium DPD excluded any bone involvement.

The histological examination of the breast masses reported aggregates of foamy macrophages, scattered touton (giant) cells, admixed with small lymphocytes and fibrous tissue, and focal hemosiderin depositions. 

The findings of migration and infiltration of lipid laden CD68+/Cd1a(-) histiocytes confirmed the diagnosis of ECD.

The molecular pattern BRAF v600e was not present. 

**Figure 6:**
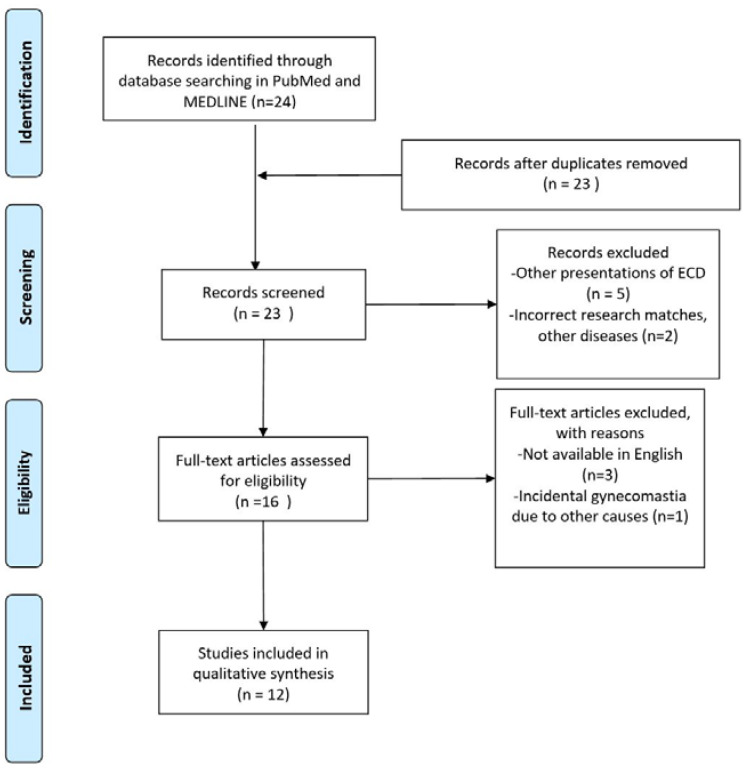
PRISMA flow diagram.

No other cases of gynaecomastia without skeletal or systemic involvement have been found in the English scientific literature. A total of 24 articles were found. Among these articles, only 12 met the inclusion criteria. The flowchart is presented in Figure 6. The selected manuscripts were published between 1995 and 2017, ten of them being edited from 2003 onwards. Ten cases have been reported from 2014. We added to the review our case report. The total amount of patients included was therefore 16. The results are briefly described in Table 1. The mean age of the patients was 50.88 years old (range: 32–78 years old, SD: 11.82), with a high predominance of female patients (13: 81.3%). The present study contains the first and only case of isolated gynecomastia as a unique feature of Erdheim–Chester disease. Only eight patients (50%) presented bone involvement, and 100% of these had at least femoral or tibial involvement. Among female patients, ten (76.9%) had unilateral breast involvement, with the tendency for a single or multiple nodular presentation, and four of them had concomitant weight loss and/or fatigue. Among male patients, three (100%) had gynecomastia with a heterogeneous enlargement of both breasts. Cutaneous breast ulcerations and scattered microcalcifications in the breast parenchyma were present in two patients. In the other male patient, a histological examination of breast parenchyma was not performed and/or reported, but an ECD-related hyperprolactinemia was described. In the two patients in which CD68+/Cd1a(-) histiocytes were found in the breast, the gynecomastia was described to have been onset since the age of 50, with a progressive development of severe gynecomastia during the VI decade of life. Globally, 14 patients (87.5%) had breast signs as the first manifestation of ECD.

## 4. Discussion

### 4.1. Case Report

Erdheim–Chester disease is a rare multisystem pathology that consists the infiltration of histiocytes that can potentially colonise any tissue and organ.

The most frequent involvement is in the skeletal system, while the mammary one is rather rare. 

It is not currently established whether there is a trophism of the histiocytes towards any specific type of organ. Consequently, specific markers that are able to predict the disease involvement of a particular tissue are currently unavailable. The isolated involvement of the breast in a male patient has never been reported in the English literature.

The patient reported in the present study came to our attention solely because of gynecomastia, and once the diagnosis of Erdheim–Chester disease was confirmed, no other clinical manifestation of that pathology was evident; thus, the case reported in the present study can be considered the first and only case of gynaecomastia as a single clinical and instrumental feature of Erdheim–Chester Disease. The finding of lung cancer in this case can be considered merely an incidentaloma, as well as a disturbing factor in the diagnostic process.

The peculiar and severe gynaecomastia reported was challenging to treat surgically, as a clean dissection was almost impossible to perform. The need for a radical excision of both mammary glands in order to restore harmony and balance to the patient’s body, as well as for the return to a normal working and sexual life, led us to perform a subcutaneous mastectomy with a single inframammary scar and a free nipple-areolar complex transplant.

This led to NAC necrosis, which did not impair the patient’s satisfaction towards the surgical operation, and resulted in a deep improvement of his psychological condition.

### 4.2. Review

Considering that, from 1930 to 2022, more than 1500 cases were published in the world medical literature or were included in the ECD Global Alliance Registry [10,48], and considering the number of reported patients in this review, the percentage of breast involvement would be close to ~0.1%; however, taking merely into account the cases reported in the global literature (~800) [49], the percentage rises to 2%.

Moreover, it is likely that this percentage again underrates the percentage of breast involvement, since 10 patients out of 16 have been reported from 2014 to 2022. 

The majority of patients reported were female (81.3%), but according to the best evidence, ECD has a male predominance (3:1 ratio; 70–75% in two different cohorts) [50,51]. 

We would suggest two different explanations of this incongruence: either (1) the Erdheim–Chester disease has a peculiar breast pattern influenced by hormones, or, more likely, (2) most of the cases of ECD-related gynecomastia remains unknown or classified as idiopathic because of the absence of other relevant features of the disease or their incorrect evaluation.

The systematic review shows that among patients with breast involvement ECD, bone involvement is much lower than as reported in the literature (50% vs. 95%). Remarkably, it is possible to identify two distinct presentation patterns between males and females: nodular in women with, in some cases (4), an association with a decline in general conditions, i.e., feelings of fatigue and weight loss; and widely infiltrating in men, up to severe gynecomastia conditions, such as the one described by us. These data require additional confirmations and, given the scarcity of reports and the lack of knowledge about the disease, it is not yet possible to formulate conclusive statements.

Most of the reports date back to a limited period of time, i.e., from 2003 until present day, which is consistent with what was stated by other authors, showing that from 2003 to 2013, there is a large increase in the number of ECD reports due to an improved awareness of the pathology, which improved the diagnostic sensibility [52]. 

This aspect, along with the high percentage of patients in which ECD was discovered following breast semeiotics (87.5%), should lead to a higher consideration for the possibility of ECD, even without other clinical features, among cases of idiopathic gynecomastia (according to Narula and Carlson [53], after the diagnostic evaluation was completed, about 25% of patients were found to have idiopathic gynaecomastia) and breast nodules that are not part of the classic pathologies, given the potential lethality of the disease and the distinct therapeutic opportunities.

## 5. Conclusions

This reported case is a unique addition to the literature and represents a diagnostic and therapeutic challenge. 

In retrospect, the diagnostic therapeutic moments were found to be preserved mainly by the plastic surgeon (who could represent this case as a generic breast caregiver) reflecting on the history described in this article, since there are no other organs involved besides the breast, which, however, remained the only true concern of the patient even during chemotherapy for lung cancer. Overall, we consider the data of our review to be unique in the literature and useful in particular to the breast unit operators, who are not always on guard against this rare, serious, and life-threatening disease [54,55,56,57] among the possible causes of isolated or idiopathic gynecomastia [58]—when ECD occurs in men—or mammary nodules—when it occurs in women. Last but not least, the aim of the study is to provide the first data as the basis for further prospective studies and reports on a disease, the Erdheim–Chester, and one of its manifestations, which has been underestimated.

## Figures and Tables

**Table 1:** Breast involvement in Erdheim–Chester disease in the English literature.

Author	Age-Sex	Location	Breast Features	Breast Involvement as First Sign of ECD	Long Bones Involvement	Relevant Medical History	Fatigue/Weight Loss
Tan et al. [23] 1995	59, F	Monolateral; left breast	Masses in the breast, with diffused soft tissue infiltration in the left mammary gland	Yes	Yes -Tibial and humeral shafts	ECD-associated lesions in pericardium, retroperitoneum, kidneys, abdominal wall	Yes
Ferrozzi et al. [30] 2000	60, M	Bilateral; widespread enlargement	The breasts began to increase in size from the age of 50 years until the onset of a giant bilateral gynaecomastia; cutaneous ulcerations bilaterally on the breasts CT: enlargement of both breasts with heterogeneous structure, microcalcifications, and foci of fatty density resulting in gynaecomastia	Yes	Yes -Both tibiae (epiphyseal osteolytic and sclerotic lesions)	Chronic pancreatitis; Leriche syndrome; antidepressant drugs; scrotal cutaneous induration and ulceration	-
Andrade et al. [41] 2003	40, F	Monolateral; Right breast; external upper quadrant	Single soft breast lump, with nodular periductal and a diffused distribution of histiocytes	No	Yes -Symmetrical diaphyseal steosclerotic lesions in both tibias and fibulas	History of Langerhans cell histiocytosis of the hard palate	-
Lemos et al. [42] 2004	49, F	Monolateral; left breast, superior to the nipple	Massive proliferation of cells with epithelial-like appearance in the breast parenchyma around malignant cells in a patient known for breast cancer (infiltrating ductal carcinoma)	Yes	No	T4N1M0 infiltrating ductal carcinoma	-
Barnes et al. [24] 2005	49, F	Monolateral Right breast; lower outer quadrant	Palpable lump; developed further breast lesion (right breast lump in the upper inner quadrant)	Yes	Yes -Osteosclerotic lesion in the distal diaphysis and metaphysis of right femur	Previous hysterectomy and bilateral carpal tunnel syndrome; diabetes mellitus; diarrhoea in the year preceding the ECD diagnosis	Yes
Provenzano et al. [25] 2010	78, F	Bilateral; lower inner quadrant (left breast); lower outer quadrant (right breast)	Suspicious 61mm spiculate mass with nipple-retraction (left breast); 69 mm soft nodularity (described as asymmetric at mammography) in the right breast	Yes	No	Hypertension; ischaemic heart disease; cerebrovascular disease with an acute pontine infarct	Yes
Furuta et al. [43] 2014	49, F	Monolateral; left breast	Well-defined round mass, 2.5 cm in diameter	No	Yes -Multiple osteosclerotic lesions in metaphysis and epiphysis of both femora and tibae	Polipectomy for a colon adenoma; allergic to alcohol	-
Loh et al. [29] 2015	55, M	Bilateral; widespread enlargement	Gynecomastia rising a few years before the clinical report	Yes *	Yes -Sclerotic lesions in both distal femura	Hepatomegaly; bilateral xanthelasma; hyperprolactinaemia and empty sella syndrome; 2.5 × 1.3 cm mass in the right atrium; congestive cardiac failure with cardiac dysrhythmia; diminished testicular volumes; hypogonadotrophic hypogonadism; hypothyroidism; secondary hypoadrenalism and GH deficiency; thickening of the pleura and of the thoracic aorta; osteosclerosis of the long bones	-
Shuangping Guo et al. [44] 2015	61, F	Monolateral; right breast	Unencapsulated yellow-grey mass, 3 × 2 cm in size. Further developed multiple masses/nodules in right breast.	Yes	Yes -Bilateral sclerotic lesions of femurs	ECD-related multiple subcutaneous nodule on the abdominal wall, bilateral sclerotic lesions on femurs, lesions on cerebellum	-
Basara et al. [45] 2015	62, F	Bilateral breast masses in the upper outer quadrant	Palpable breast masses in the upper outer quadrant of both breasts and axillar regions	Yes	No	Xanthomatous granuloma on eyelids 6 years before	-
Roverano et al. [46] 2016	I. 38, F; II. 32, F; III. 38, F; IV. 35, F	Patient I and II: left breast Patient III and IV: right breast	Erythematous and painful breast masses (size ranging 0.18 × 0.05 to 3 × 5 cm)	Yes	No	Not specified	-
Binyousef et al. [47] 2017	52, F	Bilateral; multiple breast and axillary swellings	Large hard breast and axillary masses	Yes	Yes -symmetrical sclerosis of distal femurs and tibias	Diabetes insipidus	Yes
Present report	57, M	Bilateral, widespread asymmetric enlargement	The breasts began to increase in size from the age of 50 years old until the onset of a giant asymmetric bilateral gynaecomastia; cutaneous ulceration on left breast; mammography showed parenchymal simple cysts, with dense fibroglandular breast; intraoperative findings of scattered microcalcifications	Yes	No	Lung cancer incidentally discovered during pre-operative imaging exams for mastectomy	-

* The patient reported gynecomastia due to, according to the authors, hyperprolactinemia related to an empty sella syndrome (ESS). This patient was included in our study because it was an ECD-related ESS; thus, the gynecomastia is a breast clinical feature of ECD. Further considerations can be found in the manuscript.

## Data Availability

No new data were created or analyzed in this study. Data sharing is not applicable to this article.
